# Origin of Cancer: Cell work is the Key to Understanding Cancer Initiation and Progression

**DOI:** 10.3389/fcell.2022.787995

**Published:** 2022-03-01

**Authors:** Rainer G. Hanselmann, Cornelius Welter

**Affiliations:** IB-Cancer Research Foundation, Saarbruecken, Germany

**Keywords:** carcinogenesis, cell work, energy, cell mechanics, microenvironment

## Abstract

The cell is the smallest unit of life. It is a structure that maintains order through self-organization, characterized by a high level of dynamism, which in turn is characterized by work. For this work to take place, a continuous high flow of energy is necessary. However, a focused view of the physical relationship between energy and work is inadequate for describing complex biological/medical mechanisms or systems. In this review, we try to make a connection between the fundamental laws of physics and the mechanisms and functions of biology, which are characterized by self-organization. Many different physical work processes (work) in human cells are called cell work and can be grouped into five forms: synthetic, mechanical, electrical, concentration, and heat generation cell work. In addition to the flow of energy, these cell functions are based on fundamental processes of self-organization that we summarize with the term Entirety of molecular interaction (EoMI). This illustrates that cell work is caused by numerous molecular reactions, flow equilibrium, and mechanisms. Their number and interactions are so complex that they elude our perception in their entirety. To be able to describe cell functions in a biological/medical context, the parameters influencing cell work should be summarized in overarching influencing variables. These are “biological” energy, information, matter, and cell mechanics (EMIM). This makes it possible to describe and characterize the cell work involved in cell systems (e.g., respiratory chain, signal transmission, cell structure, or inheritance processes) and to demonstrate changes. If cell work and the different influencing parameters (EMIM influencing variables) are taken as the central property of the cell, specific gene mutations cannot be regarded as the sole cause for the initiation and progression of cancer. This reductionistic monocausal view does not do justice to the dynamic and highly complex system of a cell. Therefore, we postulate that each of the EMIM influencing variables described above is capable of changing the cell work and thus the order of a cell in such a way that it can develop into a cancer cell.

## 1 Introduction

Since the discovery of oncogenes and tumor suppressor genes, modern cancer research has assumed that gene mutations alone are responsible for the initiation and progression of cancer (somatic mutation theory; Weinberg, 2014). Although this theory has been considered the central dogma of carcinogenesis for almost 40 years, no proof has yet been provided that it is causal for all malignant tumors (Soto and Sonnenschein, 2004; Soto and Sonnenschein, 2011; López-Lázaro, 2018; Supplementary Material S1). We assume that mutations are not conditio-sine-qua-non, but that they are sufficient, but not necessary, for cancer cells to develop. However, if one places the cell work with the four overarching influencing variables proposed by us (energy, matter, information, and mechanics = EMIM) at the center in order to describe and understand the processes of life, a mental model emerges that makes it possible to comprehend life mechanisms and the complex processes of initiation and progression of cancer. This study aims to present and discuss this model, and we hope that broadening our perspective will contribute to the understanding of how cancer develops and to the development of new therapeutic options.

In 2016, we published a hypothesis on the cause of cancer (Hanselmann and Welter, 2016), in which we attempted to clarify two things:

(1) Mutations cannot be considered the sole cause of cancer initiation (see Supplementary Matrial S1).
(2) As an equal cause of the initiation of cancer cells, changes in the energy balance (energy) or the composition of the intra- and extracellular molecules (matter/milieu) can be responsible in addition to certain mutations in cancer genes. In the publication, we discussed that cells must essentially be regarded as complex systems that are determined by three influencing variables: energy, matter, and information*.*

In the past few years, we reviewed and evaluated the literature on this topic, and we reformulated the individual aspects. In this context, it became clear that in addition to the aforementioned variables, mechanics (cell mechanics) must also be one of the essential overarching influencing variables for the proper functioning of the cell, and this was included in this present hypothesis.

Furthermore, it became apparent that cell work should be at the center of the observation and evaluation of cells. In our view, this biophysical parameter is suitable for describing cell functions and examining and explaining them in the context of biological systems, such as functional substructures, organelles, and cells. Against this background, we will discuss that cellular work is not only influenced and possibly disturbed by one variable, namely genes (information) and their derivatives, but also by other factors, such as energy, matter/milieu, and mechanics. This approach makes it clear that the cause of cancer can also be initiated and driven by parameters other than genes.

### 1.1 Cell Mechanics

In our previous paper on this topic, we only discussed energy, matter, and information as overarching influencing variables (Hanselmann and Welter, 2016). Based on our results and those of other research groups, we consider it necessary to also name mechanics (cell mechanics, molecular mechanics, protein mechanics) as important influencing variables (Huang and Ingber, 2005; Paszek et al., 2005; Bao, 2009; Tilghman et al., 2010). Thus, Chartier et al. (2021) were only recently able to show that the hydraulic instability of a cell alone directly determines its apoptosis. There is also evidence that mechanical forces can influence enzyme activity (Bao, 2009). Still, other publications indicate that the stiffness of a surface directly influences the proliferation, vitality, differentiation, and motility of cells. Huang and Ingber (2005) even considered extracellular mechanics as a trigger for the malignant derailment of cells. On the other hand, it has already been shown in several publications that tumor cells plated on Matrigel or similar coatings begin to differentiate and transition to a “normal” proliferation rate (Donovan et al., 2001; Elster et al., 2013; Keeratichamroen et al., 2018). However, if cells have a higher proliferation rate due to their mutations and dedifferentiate, why should they revert both properties only by growing on a soft surface? In another study by Ricca et al. (2018), breast carcinoma cells growing as agglomerates were converted into normal tubular growing cells by a pressure pulse (Ricca et al., 2018). The TOFT theory also assumes that the cause of cancer development is to be found in the disturbed interaction/anchoring between stroma and epithelium (Soto and Sonnenschein, 2004; Soto and Sonnenschein, 2011). From our point of view, this assumption makes sense because for a cell, its suspension in space is crucial (LaFlamme et al., 2018). The further it moves away from its “natural” attachment pattern, the more impossible it becomes for it to perform its physiological tasks and engage in regular metabolism (Butcher et al., 2009; Schwartz et al., 2017). Cell division is greatly influenced by mechanical conditions (Minc et al., 2011). For these reasons, it was necessary to identify cell mechanics as one of the crucial EMIM influencing variables.

## 2 Origin of Cancer and Cell Work

### 2.1 Biological Systems

The system is composed of several individual parts. Ludwig von Bertalanffy defined systems in the middle of the last century as interactive relationships that are distinct from their environment, which in turn consist of other interactive relationships (Bertalanffy, 1950). Based on this assumption, systems can be understood as self-organizing functional units that are made up of different components with different properties and that can be considered as a collective whole on the basis of certain ordered mutual relationships. Based on these considerations, efforts have been made in a wide range of disciplines (e.g., meteorology, psychology, sociology) to identify and describe systems. In the field of biology, this has developed into the research area of systems biology. It is defined as the quantitative analysis of the dynamic interactions between the components of a biological system with the aim of understanding the behavior of the system as a whole and enabling predictions. For this purpose, mathematical concepts are applied to biological systems. To maintain a close connection with biology, an iterative process takes place between laboratory experiments and computer modelling (Miczka, 2008). In recent years, systems biology has made great strides, contributing to a better understanding of cell biology (e.g., metabolism) and the development of therapeutics (Kitano, 2004; Nielsen, 2017; Turanli et al., 2021). “However, system biology, at its core, is not a set of computational and mathematical techniques; these are mere tools, incredibly useful, but secondary. The heart of systems biology is simple: explaining how a system works requires an integrated outlook. For any phenotype—molecular, macroscopic, or ecological—a set of interrelated factors exist that contribute to this phenotype. Since these factors interact, they need to be studied collectively, not merely individually.” (Hillmer, 2015). Therefore, the aim of systems biology is to understand biological systems in their entirety on the basis of their components.

**FIGURE 1:**
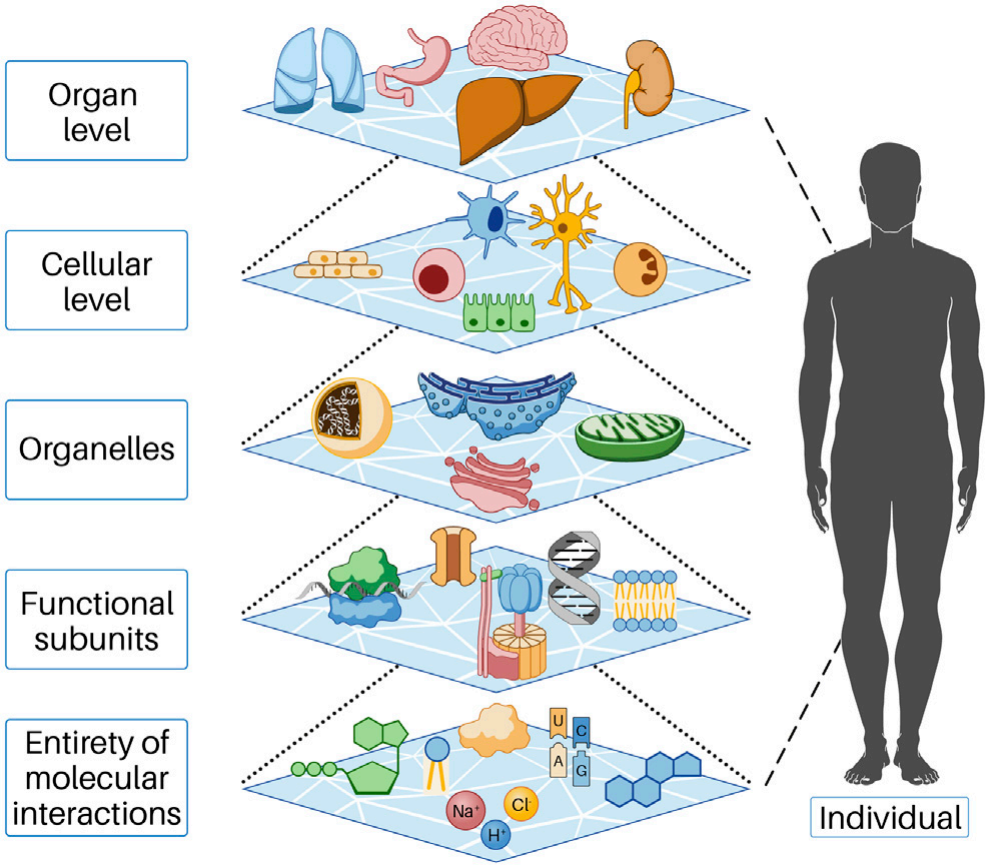
In the cell, functions occur as a result of the collective action of many molecular parts interacting and reacting with each other. These collective properties (emergence) are an essential feature of biological systems, as understanding the individual parts alone is insufficient to understand or predict system behavior. Emergent properties thus necessarily arise from the interactions of the individual parts of the system under consideration. Each of the levels shown above represents the individual parts of the next higher level. This behavior is based on mechanisms of self-organization and is only possible because energy flows permanently into the open system, predominantly as chemical energy.

Biological systems consist of a large number of components, between which there are relationships and interactions. We refer to the lowest level of these components for our consideration as the entity of molecular interaction (EoMI); it includes all molecules, ions, and other substances that interact with each other in the cytoplasm or nucleus and with the cells in their immediate environment (more on this below). The properties of a biological system are characterized by the properties of the components; thus, the system acquires properties that go beyond the sum of the properties of the individual components. This phenomenon is called emergence. In the cell, many functions result from the collective behavior of many molecular parts interacting and reacting with each other. These collective properties (emergence) are an essential feature of biological systems, as understanding individual parts alone is not sufficient to understand or predict system behavior. Thus, emergent properties necessarily arise from the interactions of the individual parts of the system under consideration. This conceptual model can be found at all levels of life, that is, beginning at the molecular level and functional substructures through the cell and organ level to the entire human being and beyond (Figure 1). In order to understand the influences responsible for the initiation and progression of cancer, we found it helpful to describe cell systems through cell work. In our view, this is necessary because cell work encompasses all cellular functions. For a more detailed explanation of cell work, see below.

### 2.2 Entirety of Molecular Interaction

The entity of molecular interaction (EoMI) encompasses all variables that fundamentally generate cell work and is thus to be understood as the totality of all molecular mechanisms, reactions, and interactions that take place inside and outside a cell, thus making the living functional cell unit possible in the first place. Because the EMIM influence quantities summarize the essential components of the EoMI, they are suitable for describing the resulting cell work (see below).

Understanding life requires knowledge of all molecular interactions and the parameters that influence them. To date, only a few of these are known. There are several reasons for this. The unmanageably high number of molecules and ions involved makes it impossible to fully understand the interplay of individual reactions. In addition, the mechanisms and interactions in the cell do not exclusively follow predetermined and determined processes and reaction cascades but are to a large extent characterized by random and stochastic events (Balazsi et al., 2011). These events are significantly influenced by the composition of their immediate environment, energy, and biophysical mechanisms (Uversky, 2017; Kerr et al., 2019). We refer to this level of life as the entity of molecular interaction (EoMI), and it can neither clearly predict how tissue cells respond to a particular stimulus nor why synchronized, genetically identical cell clones in the same microenvironment respond to the same stimulus with different cell work processes because of its complexity (Balázsi et al., 2011; Loewer and Lahav, 2011). Although many of these mechanisms have already been studied and are well understood, this world is still largely unknown. The fundamental problem lies in the complexity of the system, and for this reason, we consider it necessary to look at the cell work. It results from the EoMi interactions and processes of self-organization (Davis et al, 2013) and shows emergent properties that can be measured, observed, and put into context with other cellular working processes or systems.

To clarify what we mean by the variability, dynamics, and effects of EoMi, we present the following findings, which we assume, show that cell functions are not exclusively genetically determined, but are a consequence of the aforementioned molecular interactions, reactions, and biophysical influences that act on the molecules in the immediate environment and make it difficult to predict their function:

(1) In addition to its telomere-extending function, human telomerase (hTERT) can also function as a reverse transcriptase and an RNA-dependent RNA polymerase (RDRP) (Machitani et al., 2020; Yasukawa et al., 2020). The fact that an enzyme can perform such different functions is remarkable and raises the question of how these functions are regulated.
(2) Glycolysis enzymes are another example of this. There is increasing evidence that most glycolytic enzymes are deregulated in cancer cells and play an important role in tumorigenesis. Recent studies have shown that all essential glycolytic enzymes are translocated to the nucleus, where they are involved in tumor progression, regardless of their recognized metabolic function. These non-classical properties include anti-apoptotic functions, regulation of epigenetic modifications, modulation of transcription factors and cofactors, a role as extracellular cytokines, a protein kinase activity, and involvement in the mTorc1 signaling pathway (Kim and Dang, 2005; Gough, 2008; Tristan et al., 2011; Gao et al., 2012; Hamabe et al., 2014; Seki and Gaultier, 2017; Yu and Li, 2017). This makes it clear that these multifaceted glycolytic enzymes not only fulfil their classic tasks but can also perform a wide variety of functions. Could the properties be regulated only by genes or is it not possible that they are caused by a local milieu and stochastic processes?
(3) In a review article, Sherr and Roberts (2004) discuss that cyclins or cyclin-dependent kinases are not strictly essential for cell cycle progression. In this paper, he present several knockout mouse experiments in which one or more of these proteins were deleted. The results showed that embryos have largely developed normally. Others died *in utero*, but not with multiple morphological changes, as expected, but in some cases with only single abnormalities.
(4) In recent years, it has become clear that transcription factors involved in transcription are intrinsically disordered proteins that have special properties (high structural flexibility, interaction with a wide variety of binding partners, etc.). These highly flexible proteins are strongly influenced by the cell milieu (Alberti et al., 2019), for example, ATP concentration, pH, or the molecular composition of the milieu, to name but a few examples (Patel et al., 2017; Garcia-Cabau and Salvatella, 2021; Mehringer et al., 2021). Because of these properties, these proteins do not act according to the classical key/lock principle but follow stochastic mechanisms (Raj and van Oudenaarden, 2008; Chubb, 2017). It follows that the simplified description of transcription by protein/protein and protein/DNA interactions is not appropriate.

Against this background, we believe it will be an exciting question to determine whether and to what extent altered EMIM influencing variables contribute to shifts in protein and cell function. That this is possible in principle is already shown by generally accepted influences such as the pH value, the milieu, and the temperature on the enzyme activity or the protein structure.

Another aspect seems important to us at this point. If proteins can perform many fundamentally different functions, it is conceivable that if the function of one protein is disrupted, this function can be (partially) compensated for by another protein. Because of their homology, some proteins in a family can substitute for each other (Sherr and Roberts, 2004). However, if proteins can perform versatile functions, this would mean that a defined protein does not necessarily have to be at its site of action, as its function can be compensated for by other proteins (Sherr and Roberts, 2004; Yoon et al., 2012). In this context, intrinsically disordered proteins may be of particular interest (Wallmann and Kesten, 2020), which are discussed in more detail below.

Acceptance of EoMi would have important consequences. First, the regulatory importance of DNA would be further put into perspective, as it is the basis of the sequence, but does not influence the ultimate functionality of its derived products. This is strongly determined by the processing, localization, composition of the milieu, binding partners, and existing biophysical influences. Second, these properties would make the cell more stable in relation to the environment. It can react more flexibly and quickly, as it is not necessarily dependent on the re-synthesis of the required proteins.

From a biological perspective, nature uses two opposing principles to create life: randomness (chaos of biomolecular interaction) and determinism (order and precise coordination) (Raj and van Oudenaarden, 2008). In the past decades, biological/medical research has been striving to understand the ordered and determined processes, which has led to an immense gain in knowledge. We are only beginning to glimpse the effects of the much larger and more decisive part, which is characterized by randomness, complexity, and chaotic principles. Especially in the case of cancer, it is clear from our point of view that it is time to look at the cell more holistically.

### 2.3 Cell work is the Central Hallmark of Life

“The biological revolution of the twentieth century completely reshaped all fields of biomedical study, with cancer research being only one of them. The fruits of this revolution were revelations of both the outlines and the minute details of genetics and heredity, how cells grow and divide, how they assemble to form tissue, and how the tissues develop under the control of specific genes. Everything that follows in this text draws directly or indirectly on this new knowledge.” (Weinberg, 2014).

Molecular biology and genetics have undeniably influenced and changed our understanding of biology. Without these disciplines and their technologies, we would have only marginal knowledge of life and nature. In medicine, they have contributed significantly to the understanding of physiology and pathophysiology in the diagnosis and therapy of diseases. In particular, our knowledge of cancer has been expanded by these areas of research.

It is generally accepted that mutations in certain genes (cancer genes) trigger cancer. In addition, these genomic changes (alteration of information) are largely responsible for cancer progression.

In the following, we explain why this assumption can only be a part of the actual process. To avoid misunderstandings, we would like to clarify at the outset that we do not dispute the ability of certain mutated genes to trigger cancer or to promote its progression. What we are saying, however, is that genes can only be partly blamed for the development of cancer.

To understand the processes of life, it is necessary to place cell work at the center of considerations and evaluations. This puts the importance of genes and their derivatives into perspective. They become part of the many parameters and influences that together contribute to the complex structure of the cell characterized by self-organization. These countless reactions (e.g., dynamic equilibrium, flow equilibrium), mechanisms and interactions that make up life, and the outcome of which fascinates and confuses us at the same time.

Through this approach, we achieve several goals at once:

(1) This view explains why the excellent arguments and experimental results of alternative cancer hypotheses and studies on carcinogenesis are also valid (Warburg theory, TOFT theory, microenvironment theory, speciation theory; see Supplementary Material S1). From our point of view, however, like mutation theory, they only describe part of the complex events. For a detailed discussion, please see Hanselmann and Welter (2016).
(2) By looking at cell work processes and their EMIM influencing variables, one obtains a holistic view of defined systems, with the chance of being able to fit them into the context of another system, perhaps even 1 day to get the whole picture. This cannot be achieved with a reductionistic view of the genes and their products in a system.
(3) This approach builds another bridge between biology and medicine and their underlying sciences of chemistry and physics.

### 2.4 Cell work and the EMIM Influencing Variables

**FIGURE 2:**
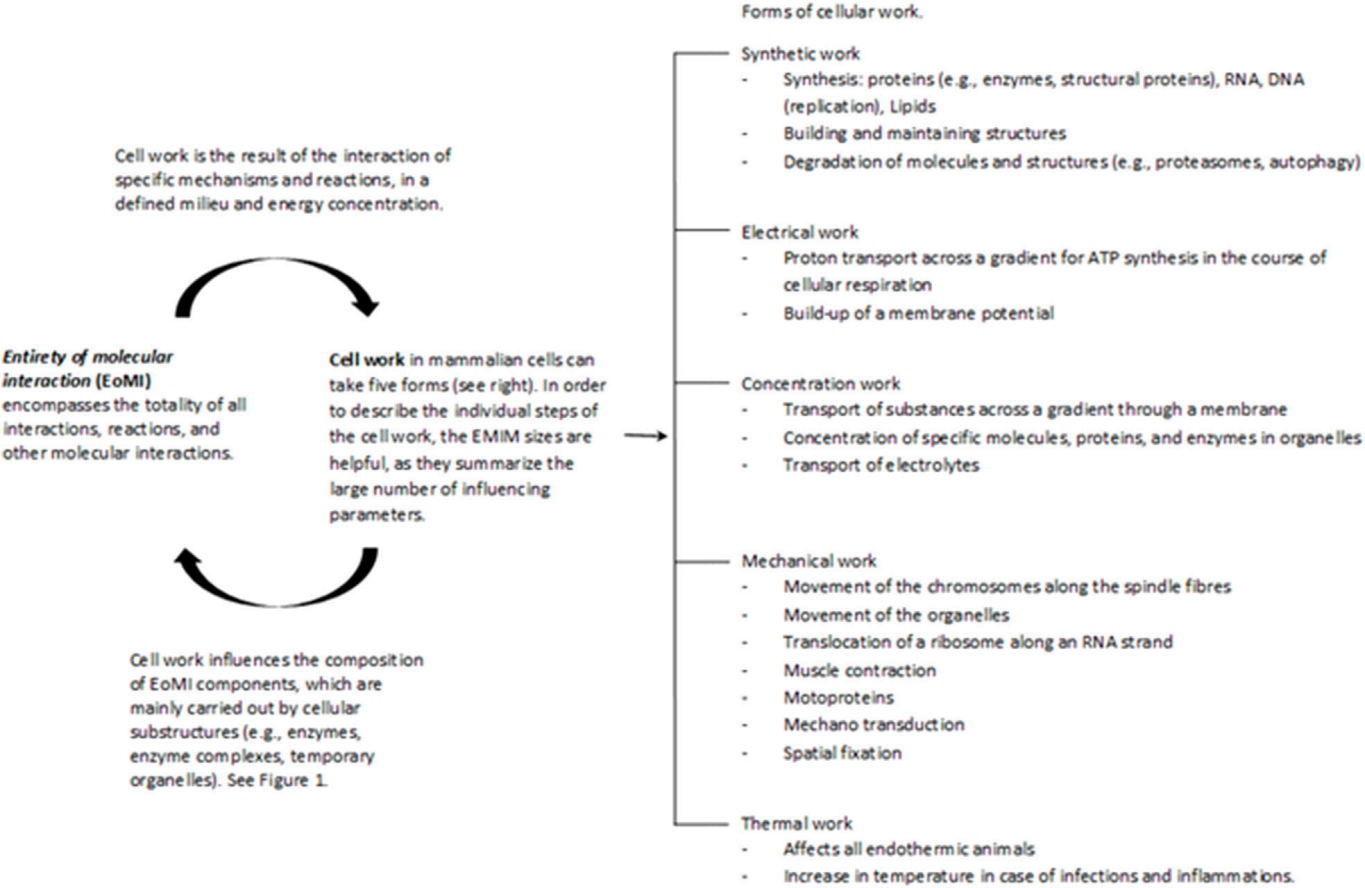
The Entirety of molecular interactions (EoMi) is characterized by a large number of very different molecules and ions reacting with each other, forming equilibriums and flow equilibriums. These mechanisms are marked by a high degree of dynamism and are characterized by self-organization. The essential driver of these processes is the flow of energy through which work/cell work emerges. This cell work can be divided into five categories under which different work processes can be grouped (see figure). What is interesting in this context is that the EoMI produces cell work through self-organization and that these in turn influence the composition of the EoMi through the various work processes. Cell work is an emergent property of EoMi. The various forms of cell work can come together to form superordinate systems that exhibit new emergent properties, which in turn influence the system (see also Figure 1).

**FIGURE 3:**
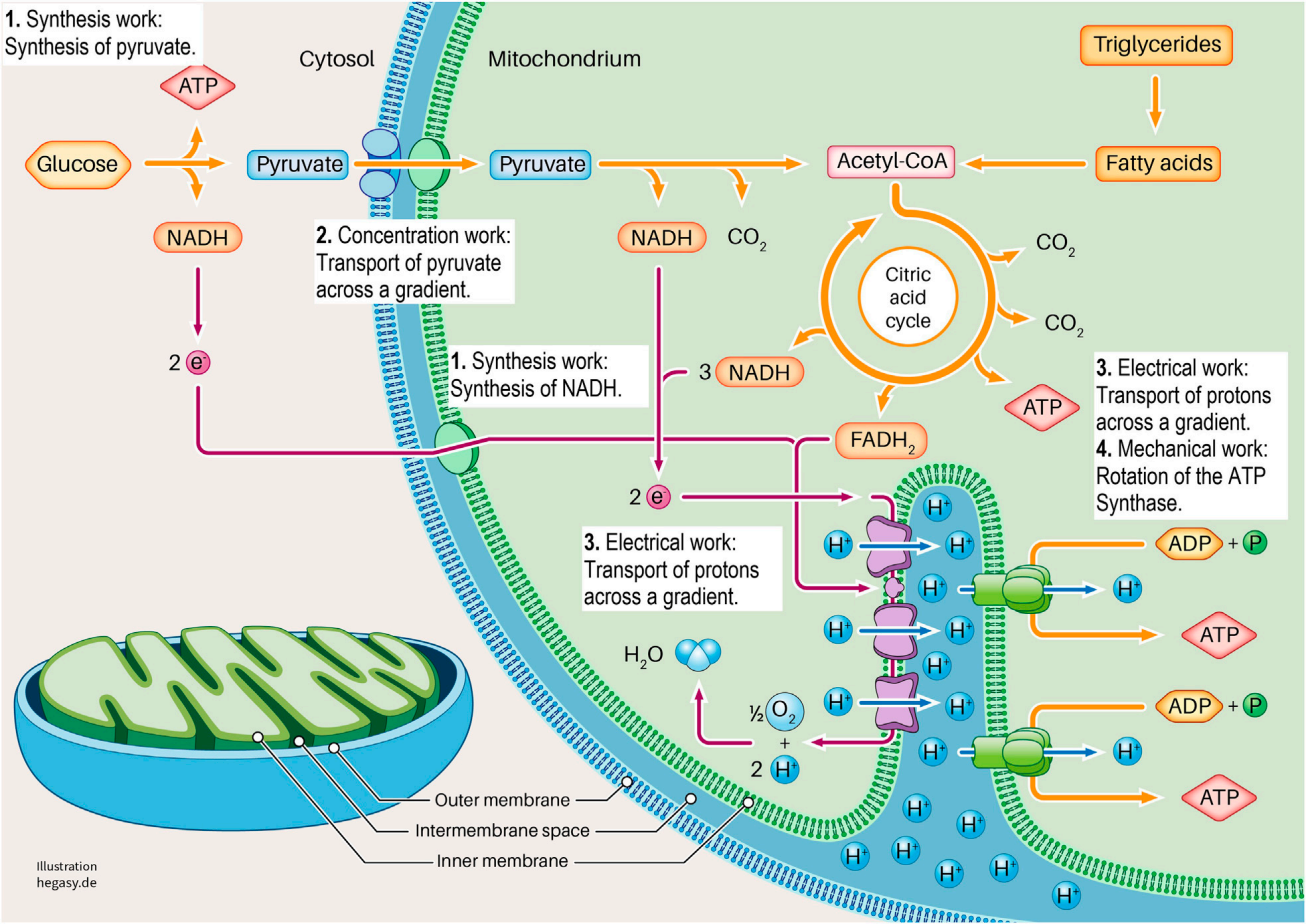
Here we show the section of a cell in which glycolysis and cell respiration are shown in a simplified form. It is clear here that this system requires four forms of cell work to generate ATP (1-4). These are synthesis work (1), concentration work (2), electrical work (3), and mechanical work (4). See the text for further explanations. To describe the individual work processes and their interrelationships in more detail, it is not sufficient to look at the individual proteins. For this purpose, further important parameters must be considered. These are the existing energy, the existing milieu, and the mechanics that influence different levels. In this way, the cell working process can be adequately characterized and described.

**FIGURE 4:**
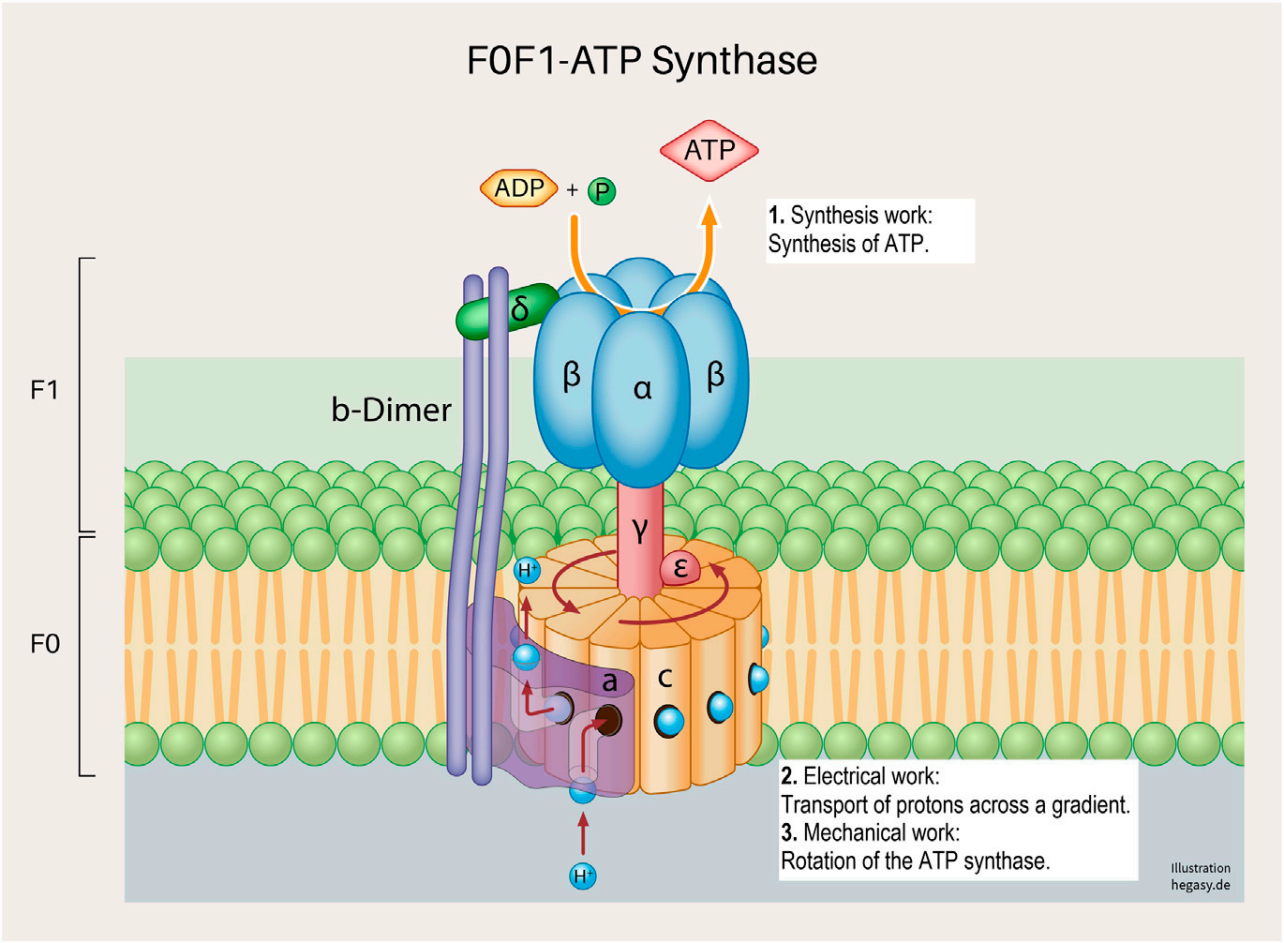
A subunit of the cell respiration described above; the F0F1 ATP synthetase system in the inner mitochondrial membrane (Neupane et al., 2019). Various forms of cell work are involved, and which are necessary for a correct process: synthesis work (1), electrical work (2), and mechanical work (3). These working processes are also, as already described above, not only influenced by the regular structure of the proteins but are also dependent on the surrounding biological energy, the milieu and mechanical influences.

Cell work is the main characteristic of life and must therefore be at the center of the consideration and evaluation of life and diseases. Please see the glossary or the corresponding specialist literature to explain the connection between energy and work. In mammalian cells, five basic forms of cell work can be distinguished (Hardin et al., 2012): synthetic, mechanical, electrical, concentration, and heat generation. These basic mechanisms influence cells and their systems to varying degrees, thus enabling cell or body functions (Figure 2). Cell work within a system can present itself in many ways and at different levels. We would like to illustrate this approach using an example of ATP production in the mitochondria (see also Figures 3, 4).

Glycolysis and the respiratory chain play a central role in ATP production. If we take a closer look at this system, we see that various forms of cell work are necessary. It begins when pyruvate is synthesized from glucose in the cytoplasm *via* glycolysis (synthetic work). Pyruvate is then actively transported into the matrix of the mitochondrium (concentration work) and contributes to the synthesis of NADH and FADH_2_ in the citric acid cycle, among other products (synthetic work). These molecules provide electrons that transfer electrons to oxygen in a complex sequence in an electron transport system (ETS). In this process, protons are transferred from the matrix into the inner membrane space, which leads to the build-up of a proton gradient between the matrix and the intermembrane space (electrical work). ATP synthase uses this gradient to produce ATP from ADP and phosphate (synthetic and mechanical work). In addition, the entire respiratory process involves the release of heat to fulfil the second law of thermodynamics.

In this example, glycolysis and the respiratory chain represent the system under consideration. Through the interaction of various forms of cell work, ATP is ultimately produced in this system.

However, it is necessary for these different forms of cellular work to be carried out. Here, we descriptively summarize the four EMIM influencing variables of the mechanisms that condition the respective cell works (see also Figures 3, 4). We would like to illustrate this using individual examples.

(1) Energy: The flow of (physical and biological) energy is the driving force of all cell work and is therefore a mandatory prerequisite for the development of life processes.
(2) Matter (milieu): For the individual reactions and processes to take place properly, all the necessary molecules (proteins, RNA, electrolytes, and other reaction partners or other milieu partners, i.e., protons) must be present in the required concentration.
(3) Information: For instance, for the proteins involved to be present correctly, it is necessary for the coding DNA to be free from mutations and for protein synthesis to proceed properly.
(4) *Mechanics:* For the synthesis of ATP from ADP and phosphate, for example, the rotation (molecular mechanics) of parts of the enzyme complex (ATP synthetase) is necessary.

Note: On closer examination, physical and biophysical factors must be included in the evaluation of the cell work and overarching systems (e.g., pressure, temperature, viscosity, diffusion, etc.). However, this would complicate the presentation of our hypothesis and is therefore not elaborated on.

The EMIM influence quantities are suitable for describing complex cell work and disturbances. Any change in one or more of the EMIM variables can disrupt the flow of cell work and trigger the initiation of cancer or drive its progression. This assumption is supported by a large number of publications in which individual EMIM influencing variables (e.g., loss of energy, change of milieu) proposed by us are considered to be causal for cancer or directly involved in its development (see Supplementary Material S1). We have discussed these points in our former publication, “Origin of cancer: an information, energy and matter disease” (Hanselmann and Welter, 2016).

In our research, we also examined systems that are involved in the hallmarks of cancer (Hanahan and Weinberg, 2011). It is striking that many of these systems, if they do not function properly, are directly involved in the development of various hallmarks of cancer (see Supplementary Material S2). As an example, we would like to mention ion channel disorders (oncochannelopathy) (Prevarskaya et al., 2010; Litan and Langhans, 2015; Stransky et al., 2016). The authors note that ion channels, for example, are crucial for signal transduction and can thus simulate mutational effects in signaling cascades when disrupted in other ways. In this context, they point out that “Any perturbation in these orderly changes resulting from ion channel(s) dysfunction would seriously impair cell proliferation, differentiation, apoptosis, and/or motility and promote the development of one or more oncochannelopathies in the form of certain cancer hallmarks.” The questions that arise from this are as follows: If the disruption of ion channels is directly involved in crucial hallmarks of cancer, why would it not trigger a cascade at the end of which a cancer cell develops? Could these disorders be also caused by one or more pathological changes in the EMIM influencing variables, such as energy deficiency under hypoxia or by a disturbed milieu in chronic inflammation?

In addition to suggesting that disturbed ion channels can initiate the development of cancer cells, we have already pointed out that altered cell mechanics can affect proliferation and alter metabolism in such a way that they contribute to tumor initiation. Huang and Ingber point out: “Thus, cancer can no longer be viewed solely as a result of dysregulation of intracellular signaling pathways. This regulatory activity of ECM mechanics puts cell fate regulation and its pathological derailment that leads to neoplasia back into the context of solid-state tissue properties.” (Huang and Ingber, 2005).

We have listed several publications in a table in Supplementary Material S2 that show a direct correlation between typical cancer markers and an EMIM influencing variable.

In this context, it is particularly interesting to consider that no driver mutations can be detected in a proportion of malignant tumors (Versteeg, 2014; López-Lázaro, 2018). Furthermore, tumors, some of which are highly malignant, show few or no mutations in childhood (Mack et al., 2014). Based on these findings, it is necessary to identify further molecular triggers of cancer initiation and progression in addition to mutations. The mechanisms presented above can play a role here.

**FIGURE 5:**
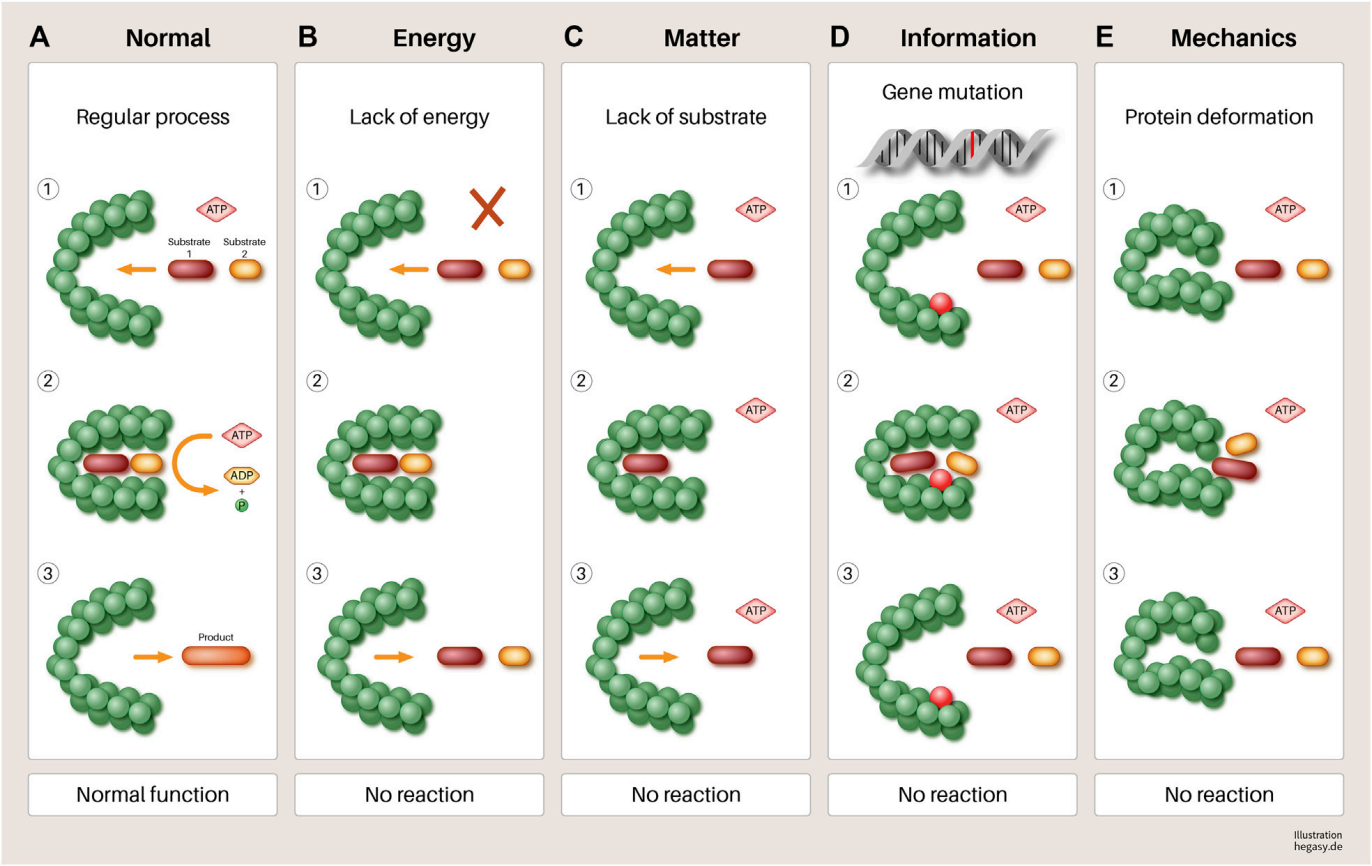
Using the example of a hypothetical protein, it is illustrated in a simple way that various influences **(B–E)** can have an impact on its function. **(A)** A normal gene is transcribed and translated. When sufficient substrates and biological energy are present, it performs its function, which leads to the required product. **(B)** lack of biological energy, **(C)** effect of mechanical forces (pressure and tensile forces), **(D)** mutation of a gene, and **(E)**. lack of substrates involved in the process lead to a failure to synthesize the required product and to the accumulation of reactants. If this disturbed mechanism persists, this leads to increasing disorganization of this reaction pathway, in the course of which, further processes can be disturbed. At the end of such a process, dedifferentiated and malignant cells could emerge. Other parameters not shown here that have negative influences are, for example, a non-physiological pH value, high temperatures, altered protein solubility due to low ATP concentration, overcrowded milieu, non-physiological electrolyte concentration, or other molecules of the environment. In addition, substances that are not endogenous to the body (e.g., toxins) can directly influence the function of enzymes that lead to irreversible changes in the metabolism of the cell without mutations.

However, it seems doubtful that the mutations of corresponding cancer genes alone lead to the disruption of cell work in systems and thus, to cancer, especially if one takes into account that the same or a similar effect can be brought about by changes in another EMIM influencing variable (see Figure 5).

## 3 The Role of the Genome

Genetic information is stored in the DNA which is a data storage device that is primarily passive. Therefore, everything that happens to the DNA is determined externally: transcription, replication, recombination, repair, and epigenetic modification mechanisms. All these processes are not direct properties of the genome but are carried out by molecules that are in the environment of the DNA and are conditioned by the interaction of the molecules in the cytoplasm.

In other words, the passive genome is surrounded by a wide variety of molecules that combine into specific structures through self-organization in order to read information from the DNA or to change it in the sense of the processes taking place in the cell nucleus and cytoplasm, i.e., to use it or to pass it safely from generation to generation. We would like to demonstrate this connection with the following example of epigenetics:

### 3.1 Example of Epigenetics

“An epigenetic trait is a stably heritable phenotype resulting from changes in a chromosome without alterations in the DNA sequence.” C. H. Waddington (Berger et al., 2009).

“For decades, it was thought that the only heritable information transmitted from one individual to another was encoded in the DNA sequence. However, it has become increasingly clear that this is not the case and that the transmission of molecules from within the cytoplasm of the gamete also plays a significant role in heritability.” (Frolows and Ashe, 2021).

Classical epigenetics basically deals with the old question of whether all living beings are pre-programmed by their genes or whether, and if so, to what extent are they changed and shaped by their environment? After Watson and Crick decoded the structure of DNA, there has been rapid development in the fields of molecular biology and genetics. In 1970, Crick proposed that the information stored in the DNA can be transcribed into the RNA if necessary and then translated into a protein (dogma of molecular biology/central dogma). The possibility of reversing this process was excluded from the theory (Crick, 1970). This developed into the generally accepted view that all processes of the cell are controlled and organized via this mechanism. In the field of heredity, it soon became apparent that this assumption could not be true (Loison, 2021) since certain traits were transmitted from parent to child without the genome showing any corresponding changes. This explanation lies in the epigenetic processes of cells. Various molecular mechanisms, influenced by external circumstances, ensure that genes are more or less strongly replicated in subsequent generations without changing the information stored in the DNA sequence (Waddington, 1942). Three molecular epigenetic processes can be distinguished: modification of chromatin and DNA methylation and regulation by small interfering RNA molecules (siRNAs) (Ashe et al., 2021). According to Ashe et al., certain traits can be inherited, even independent of the genome. For example, the siRNAs mentioned above are involved in epigenetic inheritance (Anderson and Kedersha, 2009; Wei et al., 2017).

Epigenetics can also be considered as a system. Various forms of cell work lead to the inheritance of traits without the need to change the sequence of the genome. It is important to note that in the context of this publication, we refer to epigenetic inheritance, that is, the inherited traits transmitted during mitoses (Ashe et al., 2021). We have already pointed out that classical epigenetics is not limited to the modification of DNA, but that siRNAs, for example, also contribute to the inheritance of traits, independent of DNA modification. We assumed that inheritance occurs to a far greater extent without genomic involvement. For example, several groups described a protein driven inheritance in different cell types including eucaryotes (Chakrabortee et al., 2016; Harvey et al., 2018).

However, during cell division, the DNA with all its modifications is replicated in the S phase and later distributed equally. Even before replication begins, all other cellular components are also replicated. It is generally accepted that their molecular structure not only corresponds to the genomic template but is also altered and modified by a variety of different steps (Alberts et al., 2011). These changes are individual to each cell, and they represent, in aggregate, the adaptations that each individual cell must make in order to function properly in a specific environment (Raj and van Oudenaarden, 2008). They are a result of the cellular environment, the molecules that have been taken up and already processed in a wide variety of ways, the energy available, and the mechanical barriers that allow reactions to take place in a limited space. These countless individual reactions and modifications are significantly influenced and controlled by the local conditions.

This has consequences for heredity. If each cell has an individual composition and active cellular working processes (Balázsi et al., 2011; Loewer and Lahav, 2011), the newly synthesized molecules must correspond, in their modifications, to those of the individually adapted existing molecules. This ensures that the genomic, nuclear, and cytosolic composition of the daughter cells is as similar as possible to that of the mother cell and that they are optimally prepared for the existing environmental milieu.

Against this background, we also assume that individual changes will continue to increase over the course of life. This means that in addition to genomic aging (= increasing mutation rate, Yokoyama et al., 2019), a cell also ages metabolically or, if you will, the entity of molecular interaction (EoMI). If we consider the number of substances that humans ingest in the course of their lives (nutrients, salts, water, strong and toxins, inert, lipophilic, hydrophilic substances, etc.) and the stressors to which tissues are exposed (hypoxia, glucose abuse, metabolic disorders, inflammation, infections, radiation, etc.), it becomes clear what external influence cells are subjected to, and to which they then react individually both genetically and with their molecular interactions. These influences lead to changes that permanently manifest themselves genetically, epigenetically, and at the EoMI level, continuously transforming the cell. Depending on the type of substances and the duration of exposure, the change can not only result in cancer cells in certain cases but could also be the cause of chronic diseases. We would like to emphasize here that everything our body ingests and everything that has an external effect on it fundamentally contributes to the change of our cells and our body.

### 3.2 The Complex Influence of the Variable Information on Entirety of Molecular Interaction, Cell Work, and Cancer

Although we have pointed out that genes cannot be held solely responsible for the development of cancer, we would like to show here using a few examples that mutations can influence cellular work processes and initiate cancer. This is particularly evident in inherited forms of cancer, such as hereditary retinoblastoma (RB gene) or Li-Fraumeni syndrome, in which patients with a mutation in the TP53 gene have a 50% risk of developing different tumors (Bougeard et al., 2015). Both mutations also occur in somatic tumors. Thus, penetrant mutations in the retinoblastoma (RB) gene lead to inactivation of the RB protein, which is often found in tumors (Weinberg, 1995; Dick and Rubin, 2013). The role of the RB family here is not limited to proliferative control alone but is involved in various cellular work systems, including maintenance of genomic stability, regulation of apoptosis, involvement in cell metabolism, senescence, angiogenesis, and suppression of invasion and metastasis (Dick and Rubin, 2013; Indovina et al., 2013). Mutations in this gene disrupt the order of these very different working processes, which can lead to malignant transformation.

Another regulator, p53, is the most frequently mutated cancer gene (Mendiratta et al., 2021). The protein is an important stress detector and responder that is sensitive to a variety of stress factors. These include genotoxic stress, excessive signaling, nutrient deficiency, and hypoxia. Through checkpoints in the cell cycle, p53 can arrest it, for example, to enable the repair of defective DNA or, if the damage is irreparable, to initiate cell death (Kruiswijk et al., 2015; Haupt et al., 2016). In addition, p53 is associated with the adaptation of metabolism, regulation of autophagy, and redox homeostasis (Matoba et al., 2006; Kruiswijk et al., 2015). Against this background, it can be assumed that mutation-induced modifications of the p53 protein result in changes in EoMI. As a result, certain cellular work processes change in such a way that a new cell order develops, which can lead to a tumor.

From the above examples, it is clear that the influencing variable information, as well as the other variables, can cause a susceptible imbalance in the complex interplay of cellular work processes and can significantly alter cells. Therefore, its importance in the development of cancer is undisputed.

At this point, we would like to briefly discuss the properties of the genome (information) that highlight its special position within the four EMIM influencing variables. We have described DNA as a molecule that serves exclusively to store information and is otherwise completely passive. However, this view is one-sided, because after copies of the DNA have been created in the form of RNA through the active process of transcription and the information is now available in a different form. This pathway is partly processed (e.g., splicing) and modified in various ways. In addition, a large number of different non-coding RNA molecules are formed (e.g., miRNA, piwi-RNA, sno-RNA, long non-coding RNA, tRNA, etc.). These are involved in a large number of cell processes (gene regulation, splicing, transcription, translation, post-transcriptional regulation, etc.). In recent years, it has become clear that the importance of RNA is much greater than previously thought, and we are only beginning to understand the resulting implications (Elliott and Ladomery, 2016). Information is converted into proteins through translation. According to the current state of knowledge, proteins can be modified and changed to an even greater extent. This makes their variability higher than that of RNA (Ho et al., 2018). Together, RNA and proteins account for up to 50% of the cell mass (Milo and Phillips, 2015; Uversky, 2017), which means that information and the proteins formed on its basis are of great importance in purely quantitative terms in the complex interplay of the entity of molecular interaction (EoMI). As the next chapter will illustrate, biophysical quantities have a great influence on the functionality of biomolecules, such as RNA and proteins. In addition, they constantly interact with molecules in their environment and are thus affected by their functions. Furthermore, their function is strongly dependent on other EMIM influencing variables (energy, matter/environment, and mechanics).

## 4 Examples for Biophysical Influencing Mechanisms

As mentioned above, in addition to the EMIM influencing variables, biophysical influences such as temperature, pH value, concentration, and ionic strength play an important role in all biological processes. At this point, we would like to point out two further interesting aspects that, in our opinion, have not yet been sufficiently included in the consideration and evaluation of cell functions/cell work and the development of diseases.

### 4.1 ATP as a Biological Aggregation Inhibitor

To date, most scientists have assumed that ATP only fulfils the task of storing and providing energy. Recently, it has become known that this is not the case. Obviously, the cell needs the energy transferred by ATP to carry out the reactions. However, ATP must only be present at micromolar concentrations. The intracellular concentration of ATP, however, is in the millimolar range (between 2 and 8 mM; Patel et al., 2017). Recent research has shown that ATP at this concentration acts as an aggregation inhibitor for proteins. In fact, it is considered a key molecule for protein solubility *in vivo* (Mehringer et al., 2021). That is, ATP prevents protein aggregates and is able to dissolve existing protein aggregates (Patel et al., 2017; Garcia-Cabau and Salvatella, 2021; Mehringer et al., 2021). This property sheds new light on such an important molecule. For us, this raises exciting medical questions: Are the effects observed under hypoxia the result of the lack of energy or do we find newly formed agglomerates that make it impossible for the affected proteins to function properly? What happens when the ATP concentration rises again after reoxygenation? Do all aggregates then dissolve again or do stable clusters emerge that disrupt cell metabolism? What happens in chronic inflammation and chronic circulatory disturbances?

In any case, if one wants to assign ATP to the EMIM influencing variables in the context of our hypothesis, one recognizes that it belongs to both the energy and the matter/milieu variable. On the one hand, its loss means that there is not enough energy available to carry out phosphorylation. On the other hand, its reduced concentration can alter the solubility of a protein in such a way that it changes its functionality (Somalinga and Roy, 2002) or perhaps even loses it. Both aspects can have far-reaching consequences for the cells, such as increasing disorder within a system or a work process.

### 4.2 Intrinsically Disordered Proteins, Liquid-Liquid Phase Separation, and EMIM

Eukaryotic cells are filled with a wide variety of molecules, including macromolecules of nucleotides, polysaccharides, and proteins. There is little space left for movement, diffusion, and free water (Uversky, 2019). However, the cell can ensure a regular reaction process. This phenomenon is called the crowded milieu, and the insights gained in this context have revolutionized protein research and challenged the classical sequence-structure paradigm (Schlessinger et al., 2011; Babu, 2016). An important discovery in this context was that proteins were found to be highly flexible and dynamic, the so-called intrinsically disordered proteins (Uversky, 2017; Uversky, 2019). This property arises from the fact that certain amino acid chains, due to their sequence, do not adopt a stable structure, as is generally assumed, but move incessantly in an uncontrolled three-dimensional manner. Since this discovery, attempts have been made to categorize proteins according to this property. In this publication, we would like to use the classification of Deiana et al. (2019), as some interesting medical references are also discussed under this categorization. Almost all proteins contain intrinsically disordered regions. Deiana et al. (2019) divided them into three groups for their studies: ordered proteins (ORDPs), intrinsically disordered regions (IDRPs), and intrinsically disordered proteins (IDPs; see Table 1).

(1) Ordered proteins (ORDPs) contain only a few disordered residuals. They do not carry a disordered polypeptide chain at the C- or N-terminal end that is longer than 30 amino acids, nor a disordered chain that has over 40 amino acids within the polypeptide. They do not contain disordered domains.
(2) Intrinsically disordered region (IDPRs): have less than 30% disordered residues and, at least at the C- or N-terminal end, a disordered polypeptide chain that is longer than 30 amino acids or a disordered chain that has over 40 amino acids within the chain.
(3) Intrinsically disordered proteins (IDPs): have more than 30% disordered residues in a polypeptide chain.

**TABLE 1:** Overview of the type, proportion, and function of intrinsically disordered protein*s* in the cell.

Variants	Proportion of total protein in %	Functions
ORDPs	49	e.g., Transport-, Receptor-, Catalysis proteins
IDRPs	19	e.g., Transport-, Receptor-, Catalysis proteins
IDPs	32	e.g., nucleic acid and chromatin binding proteins, transcription factors

Proportion of the different intrinsic disordered proteins in the total protein amount. In addition, key functions can be assigned to different variants (Deiana et al., 2019).

The advantage of IDPs is that they have no fixed 3D structure. This significantly broadens the spectrum of molecules and proteins that they can react with. This property allows them to interact with and react with many substrates with relatively high specificity and low affinity. This interaction is often associated with a disorder-to-order transition. This means that the moment an IDP interacts with a partner, it changes its conformation to carry out a reaction. In addition, disordered regions show many different post-translational modifications, the so-called post-translational modification sites (PTMs), which lead to conformational changes that result in a “one-lock-many-keys interaction” (Uversky, 2017). With these modifications, cells can adapt flexibly to changes (Kulkarni and Kulkarni, 2019). This contrasts with ORDPs and IDPRs, which interact to a greater extent according to the lock-and-key theory with only one partner.

Clinically interesting in this context is that IDPs are overrepresented in cancer cells compared to normal cells. However, ORDPs and IDPRs are underrepresented (Deiana et al., 2019). It seems that in this higher disorder, cancer cells rely more on dynamically adaptable proteins than on proteins that are ordered and fixed in their function. This lack of a decreasing structure is also visible morphologically by increasing dedifferentiation. Whether and to what extent these findings can already be found in the preliminary stages of the tumor and what role they play here is unknown.

IDPs can move quickly and flexibly through the cytoplasm or nucleus and adopt a specific structure when needed. It is assumed that this process is initiated or promoted by other binding partners, such as proteins, RNA, DNA, or other smaller molecules (Rhine et al., 2020). Interestingly, ATP influences the solubility of IDPs in a concentration-dependent manner (Mehringer et al., 2021). However, IDPs are also capable of another feature, namely the potential to attach to each other to form so-called biomolecular condensates (BCs; Banani et al., 2017; Uversky, 2017; Uversky, 2019) *via* a process known as liquid-liquid phase transition. BCs are diverse, and a wide variety of forms with diverse functions can be found in the literature (RNPs, p-granules, germline granules, stress granules, processing bodies, and signaling complexes among others; Uversky, 2017; Mittag and Parker, 2018; Zhao and Zhang, 2020). Thus, BCs can contain RNA, DNA, or other molecules, in addition to proteins. What they all have in common is that these cellular bodies have an even higher density (over-crowded milieu) than their full surroundings (crowded milieu) and that they are not a disordered collection of components but a kind of microreactor that accelerates certain biological reactions (Uversky, 2017; Uversky, 2019). This field of research is still in its infancy, and it is generally assumed that it will have a decisive influence on our understanding of cell processes.

In fact, initial work suggests that BCs play an important role in cancer initiation. Recently, Gupta et al. (2017) reported that the formation of stress granules may be involved in the regulation of breast cancer initiation. In this case, a key component of this complex, the G3BP2 protein (GTPase-activating protein (SH3 domain)-binding protein, seems to play an important role.

With regard to liquid-liquid phase separation, another important point is that it is highly dependent on various chemical and physical influences (Uversky, 2009; Uversky, 2017). Thus, the concentration and composition of the molecules involved (proteins, nucleic acids, salts, and others) play a decisive role in the formation and density of the various condensates. The concentration of ATP as an aggregation inhibitor for proteins must also be considered (see above). In addition, pH and temperature are of great importance (Shin and Brangwynne, 2017; Alberti et al., 2019). Furthermore, a gene mutation can lead to altered protein and structural behavior. However, what matters to us at this point is the influence of these parameters. Each of these factors can be altered in precancerous lesions or tumor tissues and are associated with cancer development or tumor progression. These are found in hypoxia, acidosis, malnutrition, intoxication, to name a few. If so, transcription (Capp, 2005), the formation of RNPs (Mittag and Parker, 2018) or other BCs, would be disrupted by phase separation shaped “microreactors.” This must have direct consequences for metabolism and other cell functions. We believe that these disturbances directly alter one or more of the cell’s working capacities irreversibly, with the result that in extreme cases, the cell can degenerate into malignancy. Future experiments investigating this functional relationship would help to clarify this matter.

In addition to the aforementioned variables, it can also be assumed that certain toxins or other stressors can influence this process. Changes in the milieu due to chronic or acute inflammation, trauma, radiation, and viral infections can also have an influence.

From the above arguments, it can be seen that the EMIM influencing variables can also influence IDPs, for instance, the influence on the structure through mutations (information) or changes in solubility (Mehringer et al., 2021) depending on the ATP concentration (matter/environment). Molecular mechanics seems to play a role in the disorder-to-order transition during contact with the reaction partners mentioned above. Another interesting aspect in this context is the influence of crowded milieu in cells. As early as 2002, Somalinga and Roy showed that the functionality of an enzyme could be reversed from a protease to a ligase if it was examined in the physiologically crowded milieu instead of in an aqueous buffer solution (Somalinga and Roy, 2002; Kuznetsova et al., 2014). Although conditions such as those found in aqueous buffer solutions are not found in cells, we consider these findings to be remarkable, as it shows that the crowding state of a cell alone has an effect on the functionality (cell work) of proteins. Whether this effect is significant in pathological processes can be postulated, since crowded milieus have an influence on phase separation, which is associated with the development of Alzheimer’s disease (Shin and Brangwynne, 2017).

## 5 Summary

Cell work is a distinguishing feature of cells. It gives the cell shape and function, generates the energy it needs, and produces progeny to ensure its continuation. The continuous changes produced by cell work necessitate a constant influx of biological energy. To date, molecular biology has suggested that the processes required for this are primarily regulated by genes. This view does not do justice to the complex events that take place; cell work can be described by four overarching EMIM influencing variables. These are matter, information, mechanics, and energy. Cell work is based on the mechanisms and processes of the entity of molecular interactions, which are characterized by complex molecular interactions and biophysical influences.

To understand the mechanisms of cancer development, it is necessary to examine the defined systems. Such systems can be organisms, cells, organelles, or a single reaction pathway. To evaluate the characteristics of a system, it is useful to know the cell work that contributes to the system. This can be a single or multiple work process, depending on the system under consideration, and should be the subject of future research.

Therefore, any disturbance of cell work can be described by the EMIM quantities, making several scenarios identifiable:

(1) The change is reversible.
(2) The change is irreversible, depending on the extent, which leads to two events:
  • The affected areas are permanently changed and lead to a permanent shift in the affected mechanisms, and as a result, dependent processes must adapt appropriately, which can lead to further changes. As further influences constantly act on the cell in the course of life, it is modified further and further. In particular, these changes can lead to the development of chronic diseases or cancerous cells.
  • Severe changes lead to cell death.

## Data Availability

The original contributions presented in the study are included in the article/Supplementary Material, further inquiries can be directed to the corresponding author.
