# Supplementary material for: Origin of Cancer: Cell work is the Key to Understanding Cancer Initiation and Progression
Source: Front Cell Dev Biol. 2022 Mar 1;10:787995. doi: 10.3389/fcell.2022.787995 (PMC8921603; doi:10.3389/fcell.2022.787995)
Supplement: Supplementary file 1 [file DataSheet2.pdf]

Supplement 2: Influence of different forms of cellular work on hallmarks of cancer.

Hallmarks of cancer cells	Synthesis/degradation catalysis	Concentration work	Electrical work	Mechanical work
**Immortality**	Cagatay Gunesb, Alush Irene Avilaa, K. Lenhard Rudolpha. **Telomeres in cancer.** Differentiation 99, 41–50 (2018). https://doi.org/10.1016/j.diff.2017.12.004<br><br>Lifeng Xu, Shang Li, Bradley A Stohr. **The Role of Telomere Biology in Cancer.** Annu Rev Pathol Mech Dis 8, 1, (2012). DOI: 10.1146/annurev-pathol-020712-164030	Natalia Prevarskaya, Roman Skryma, Yaroslav Shuba. **Ion channels and the hallmarks of cancer.** Trends Mol Med., 16(3):107-21 (2010). doi: 10.1016/j.molmed.2010.01.005.<br>*Ion channels have a decisive influence on signal transmission and can simulate mutational effects in signaling cascades. They have an influence on* ***all*** *hallmarks and characteristics of cancer cells.*		
**Continued proliferation**	Weinberg Robert. **The biology of cancer.** Second Edition, Garland Science. (2014). ISBN: 978-0-0153-4219-9<br><br>Li-Hui Wanga Chun-Fu Wua Nirmal Rajasekaranb Young Kee Shinb. **Loss of Tumor Suppressor Gene Function in Human Cancer: An Overview.** Cell Physiol Biochem. 51:2647-2693 (2018). DOI: 10.1159/000495956<br><br>Gimple RC and Wang X. **RAS Striking at the Core of the Oncogenic Circuitry**. Front. Oncol. 9:965. (2019). doi: 10.3389/fonc.2019.00965<br><br>T. Zhan, N. Rindtorff and M. Boutros. **Wnt signaling in cancer.** Oncogene 36, 1461–1473, (2017). doi:10.1038/onc.2016.304	Donna Dang, RajiniRao. **Calcium-ATPases: Gene disorders and dysregulation in cancer.** Biochimica et Biophysica Acta (BBA) - Molecular Cell Research, Vol. 1863, 6, Part B, 1344-1350 (2016) https://doi.org/10.1016/j.bbamcr.2015.11.016<br><br>Litan A and Langhans SA. **Cancer as a channelopathy: ion channels and pumps in tumor development and progression.** Front. Cell. Neurosci. 9:86. (2015). doi: 10.3389/fncel.2015.00086<br><br>Litan A and Langhans SA. **Cancer as a channelopathy: ion channels and pumps in tumor development and progression.** Front. Cell. Neurosci. 9:86. (2015). doi: 10.3389/fncel.2015.00086<br><br>Swietach P, Vaughan-Jones, RD, Harris AL, Hulikova A. **The chemistry, physiology and pathology of pH in cancer.** Phil. Trans. R. Soc. B 369: 20130099. (2014). http://dx.doi.org/10.1098/rstb.2013.0099<br><br>Natalia Prevarskaya, Roman Skryma, Yaroslav Shuba. **Ion channels and the hallmarks of cancer.** Trends Mol Med., 16(3):107-21 (2010). doi: 10.1016/j.molmed.2010.01.005. Epub 2010 Feb 16.<br><br>Zoltán Pethő, Karolina Najder, Etma rBulk, Albrecht Schwab. **Mechanosensitive ion channels push cancer progression.** Cell Calcium, Vol. 80, 79-90. (2019). doi.org/10.1016/j.ceca.2019.03.007	Chiara Galber, Manuel Jesus Acosta, Giovanni Minervini and Valentina Giorgio. **The role of mitochondrial ATP synthase in cancer.** Biological Chemistry, 401:11, 1199–1214 (2020). doi.org/10.1515/hsz-2020-0157<br><br>Laura Stransky, Kristina Cotter and Michael Forgac. **The Function of V-ATPases in Cancer.** Pysiol. Rev. Vol. 96, 3, 1071-1091 (2016). doi.org/10.1152/physrev.00035.2015	Sneeggen M, Guadagno NA and Progida C. **Intracellular Transport in Cancer Metabolic Reprogramming.** Front. Cell Dev. Biol. 8:597608 (2020). doi: 10.3389/fcell.2020.597608<br><br>Benjamin L Ricca, Gautham Venugopalan, Saori Furuta, Kandice Tanner, et al. **Transient external force induces phenotypic reversion of malignant epithelial structures via nitric oxide signaling.** eLIFE (2018). eLife 2018;7:e26161 doi: 10.7554/eLife.26161<br><br>Humphrey, J., Dufresne, E. & Schwartz, M. **Mechanotransduction and extracellular matrix homeostasis.** Nat Rev Mol Cell Biol 15, 802–812 (2014). doi.org/10.1038/nrm3896<br><br>Elster JD, McGuire TF, Lu J, Prochownik EV. **Rapid In Vitro Derivation of Endothelium Directly from Human Cancer Cells.** PLoS ONE 8(10): e77675. (2013). doi:10.1371/journal.pone.0077675<br><br>Butcher DT, Alliston T, Weaver VM. **A tense situation: forcing tumour progression.** Nat Rev Cancer. 9(2):108-122. (2009) doi:10.1038/nrc2544<br><br>Sui Huang and Donald E. Ingber. **Cell tension, matrix mechanics, and cancer development.** Cancer Cell, Vol. 8, 3, 175-176 (2005). doi.org/10.1016/j.ccr.2005.08.009<br><br>Schwartz, L., da Veiga Moreira, J. and Jolicoeur, M. (2018), **Physical forces modulate cell differentiation and proliferation processes.** J. Cell. Mol. Med., 22: 738-745. https://doi.org/10.1111/jcmm.13417
**Circumventing physiological cell death**	Weinberg Robert. **The biology of cancer.** Second Edition, Garland Science. (2014) ISBN: 978-0-0153-4219-9<br><br>Li-Hui Wanga Chun-Fu Wua Nirmal Rajasekaranb Young Kee Shinb. **Loss of Tumor Suppressor Gene Function in Human Cancer: An Overview.** Cell Physiol Biochem. 51:2647-2693 (2018). DOI: 10.1159/000495956	Donna Dang, RajiniRao. **Calcium-ATPases: Gene disorders and dysregulation in cancer.** Biochimica et Biophysica Acta (BBA) - Molecular Cell Research, Vol. 1863, 6, Part B, 1344-1350 (2016) doi.org/10.1016/j.bbamcr.2015.11.016<br><br>Litan A and Langhans SA. **Cancer as a channelopathy: ion channels and pumps in tumor development and progression.** Front. Cell. Neurosci. 9:86 (2015). doi: 10.3389/fncel.2015.00086<br><br>Natalia Prevarskaya, Roman Skryma, Yaroslav Shuba. **Ion channels and the hallmarks of cancer.** Trends Mol Med., 16(3):107-21 (2010). doi: 10.1016/j.molmed.2010.01.005. Epub 2010 Feb 16.	Chiara Galber, Manuel Jesus Acosta, Giovanni Minervini and Valentina Giorgio. **The role of mitochondrial ATP synthase in cancer.** Biological Chemistry, 401:11, 1199–1214 (2020) DOI: https://doi.org/10.1515/hsz-2020-0157<br><br>Laura Stransky, Kristina Cotter and Michael Forgac. **The Function of V-ATPases in Cancer. Pysiol. Rev.** Vol. 96, 3, 1071-1091 (2016). doi.org/10.1152/physrev.00035.2015<br><br>Evangelos Giampazolias and Stephen W.G. Tait. **Mitochondria and the hallmarks of cancer.** FEBS Journal 283 803–814 (2016) doi:10.1111/febs.13603	Cheng G, Tse J, Jain RK, Munn LL. **Micro-Environmental Mechanical Stress Controls Tumor Spheroid Size and Morphology by Suppressing Proliferation and Inducing Apoptosis in Cancer Cells.** PLoS ONE 4(2): e4632 (2009). doi:10.1371/journal.pone.0004632<br><br>Chartier, N.T., Mukherjee, A., Pfanzelter, J. et al. **A hydraulic instability drives the cell death decision in the nematode germline.** Nat. Phys. 17, 920–925 (2021). https://doi.org/10.1038/s41567-021-01235-x
**Circumventing growth suppression**	Weinberg Robert. **The biology of cancer.** Second Edition, Garland Science. (2014). ISBN: 978-0-0153-4219-9<br><br>Li-Hui Wanga Chun-Fu Wua Nirmal Rajasekaranb Young Kee Shinb. **Loss of Tumor Suppressor Gene Function in Human Cancer: An Overview.** Cell Physiol Biochem. 51:2647-2693 (2018). DOI: 10.1159/000495956	Donna Dang, RajiniRao. **Calcium-ATPases: Gene disorders and dysregulation in cancer.** Biochimica et Biophysica Acta (BBA) - Molecular Cell Research. Vol. 1863, 6, Part B, 1344-1350 (2016). https://doi.org/10.1016/j.bbamcr.2015.11.016<br><br>Litan A and Langhans SA. **Cancer as a channelopathy: ion channels and pumps in tumor development and progression.** Front. Cell. Neurosci. 9:86. (2015). doi: 10.3389/fncel.2015.00086		
**Disturbed energy balance and metabolism**	Ralph J Deberardinis. **Is cancer a disease of abnormal cellular metabolism? New angles on an old idea.** Genetics in Medicine, vol 10, 767–777 (2008)<br><br>Sinkala, M., Mulder, N. & Patrick Martin, D. **Metabolic gene alterations impact the clinical aggressiveness and drug responses of 32 human cancers.** Commun Biol 2, 414 (2019). https://doi.org/10.1038/s42003-019-0666-1		Chiara Galber, Manuel Jesus Acosta, Giovanni Minervini and Valentina Giorgio. **The role of mitochondrial ATP synthase in cancer.** Biological Chemistry, 401:11, 1199–1214 (2020) doi.org/10.1515/hsz-2020-0157<br><br>Evangelos Giampazolias and Stephen W.G. Tait. **Mitochondria and the hallmarks of cancer.** FEBS Journal 283 803–814 (2016). doi:10.1111/febs.13603<br><br>Gatenby, R., Gillies, R. **Why do cancers have high aerobic glycolysis?** Nat Rev Cancer 4, 891–899 (2004). doi.org/10.1038/nrc1478<br><br>Warburg, Otto. **"On the Origin of Cancer Cells."** Science, vol. 123, no. 3191, 1956, pp. 309–314. JSTOR, www.jstor.org/stable/1750066.	Park, J.S., Burckhardt, C.J., Lazcano, R. et al. **Mechanical regulation of glycolysis via cytoskeleton architecture.** Nature 578, 621–626 (2020). https://doi.org/10.1038/s41586-020-1998-1<br><br>Isogai, T., Park, J. and Danuser, G. **Cell forces meet cell metabolism**. Nat Cell Biol 19, 591–593 (2017). doi.org/10.1038/ncb3542<br><br>Romani, P., Valcarcel-Jimenez, L., Frezza, C. et al. **Crosstalk between mechanotransduction and metabolism. N**at Rev Mol Cel l Biol 22, 22–38 (2021). https://doi.org/10.1038/s41580-020-00306-w<br><br>Clifford P. Brangwynne, Timothy J. Mitchison, Anthony A. Hyman. **Active liquid-like behavior of nucleoli determines their size and shape in Xenopus laevis oocytes.** PNAS, 108 (11) 4334-4339, (2011). DOI: 10.1073/pnas.1017150108<br>ATP loss leads to viscosity changes of the nucleoli.
**Invasion und Metastasierung**	Fares, J., Fares, M.Y., Khachfe, H.H. et al. **Molecular principles of metastasis: a hallmark of cancer revisited.** Sig Transduct Target Ther 5, 28 (2020). https://doi.org/10.1038/s41392-020-0134-x<br><br>Ross C, Szczepanek K, Lee M, Yang H, Qiu T, Sanford JD, et al. **The genomic landscape of metastasis in treatment-naïve breast cancer models.** PLoS Genet 16(5): e1008743. (2020). doi.org/10.1371/journal.pgen.1008743	Donna Dang, RajiniRao. **Calcium-ATPases: Gene disorders and dysregulation in cancer.** Biochimica et Biophysica Acta (BBA) - Molecular Cell Research. Vol. 1863, 6, Part B, 1344-1350 (2016). doi.org/10.1016/j.bbamcr.2015.11.016<br><br>Litan A and Langhans SA. **Cancer as a channelopathy: ion channels and pumps in tumor development and progression.** Front. Cell. Neurosci. 9:86 (2015). doi: 10.3389/fncel.2015.00086<br><br>Natalia Prevarskaya 1 , Roman Skryma, Yaroslav Shuba. **Ion channels and the hallmarks of cancer.** Trends Mol Med., 16(3):107-21 (2010). doi: 10.1016/j.molmed.2010.01.005.	Chiara Galber, Manuel Jesus Acosta, Giovanni Minervini and Valentina Giorgio. **The role of mitochondrial ATP synthase in cancer.** Biological Chemistry, 401:11, 1199–1214 (2020). doi.org/10.1515/hsz-2020-0157<br><br>Evangelos Giampazolias and Stephen W.G. Tait. **Mitochondria and the hallmarks of cancer.** FEBS Journal 283 803–814 (2016). doi:10.1111/febs.13603<br><br>Laura Stransky, Kristina Cotter and Michael Forgac. **The Function of V-ATPases in Cancer.** Pysiol. Rev. Vol. 96, 3, 1071-1091 (2016) https://doi.org/10.1152/physrev.00035.2015	Janet M. Tse, Gang Cheng, James A. Tyrrell, Sarah A., et al. **Mechanical compression drives cancer cells toward invasive phenotype.** PNAS, 109 (3) 911-916, (2012). doi:10.1073/pnas.1118910109

**Genomic instability**	Elisabeth S. Wenzel, Amareshwaar T. K. Singh. **Cell-cycle Checkpoints and Aneuploidy on the Path to Cancer.** In Vivo, 32, 1-5, (2018). doi:10.21873/invivo.11197<br><br>Maciejowski, J., de Lange, T. **Telomeres in cancer: tumour suppression and genome instability.** Nat Rev Mol Cell Biol 18, 175–186 (2017). doi.org/10.1038/nrm.2016.171<br><br>Lifeng Xu Lifeng XuShang Li Bradley A Stohr. **The Role of Telomere Biology in Cancer.** Annu Rev Pathol Mech Dis 8, 1, (2012). DOI: 10.1146/annurev-pathol-020712-164030	Joshua R.Veatch, Michael A.McMurray, Zara W.Nelson, Daniel E.Gottschling. **Mitochondrial Dysfunction Leads to Nuclear Genome Instability via an Iron-Sulfur Cluster Defect.** Cell, Vol 137, 7, 1247-1258 (2009)	Evangelos Giampazolias and Stephen W.G. Tait. **Mitochondria and the hallmarks of cancer.** FEBS Journal 283 803–814 (2016). doi:10.1111/febs.13603	Takeshi Itabashi, Yasuhiko Terada, et al. **Mechanical impulses control metaphase progression.** PNAS, 109 (19) 7320-7325 (2012). DOI: 10.1073/pnas.1116749109<br><br>Fink, J., Carpi, N., Betz, T. et al. **External forces control mitotic spindle positioning.** Nat Cell Biol 13, 771–778 (2011). doi.org/10.1038/ncb2269<br><br>Nicolas Minc, David Burgess, Fred Chang. **Influence of Cell Geometry on Division-Plane Positioning.** Cell, Vol. 144, 3, 414-426 (2011). doi.org/10.1016/j.cell.2011.01.016
**Tumour-promoting inflammation**	Jennifer Kay, Elina Thadhani, Leona Samson, and Bevin Engelward. **Inflammation-Induced DNA Damage, Mutations and Cancer.** DNA Repair, 83: 102673 (2019). doi: 10.1016/j.dnarep.2019.102673<br><br>Kawanishi, S.; Ohnishi, S.; Ma, N.; Hiraku, Y.; Murata, M. **Crosstalk between DNA Damage and Inflammation in the Multiple Steps of Carcinogenesis.** Int. J. Mol. Sci. 18, 1808 (2017) doi.org/10.3390/ijms18081808<br><br>KJ O'Byrne and AG Dalgleish. **Chronic immune activation and inflammation as the cause of malignancy.** British Journal of Cancer 85(4), 473–483 (2001)	L.Munaron. **Systems biology of ion channels and transporters in tumor angiogenesis: An omics view.** Biochimica et Biophysica Acta (BBA) - Biomembranes, Vol. 1848, 10, Part B, Pages 2647-2656 (2015). doi.org/10.1016/j.bbamem.2014.10.031<br><br>Fiorio Pla A, Munaron L.. **Functional properties of ion channels and transporters in tumour vascularization**. Phil. Trans. R. Soc. B 369: 20130103 (2014). dx.doi.org/10.1098/rstb.2013.0103	Evangelos Giampazolias and Stephen W.G. Tait. **Mitochondria and the hallmarks of cancer.** FEBS Journal 283 803–814 (2016). doi:10.1111/febs.13603<br><br>Swietach P, Vaughan-Jones, RD, Harris AL, Hulikova A. **The chemistry, physiology, and pathology of pH in cancer.** Phil. Trans. R. Soc. B 369: 20130099. (2014). dx.doi.org/10.1098/rstb.2013.0099	
**Induction of angiogenesis**	Lugano, R., Ramachandran, M. & Dimberg, A. **Tumor angiogenesis: causes, consequences, challenges and opportunities.** Cell. Mol. Life Sci. 77, 1745–1770 (2020). https://doi.org/10.1007/s00018-019-03351-7<br><br>S P Balasubramanian, N J Brown, and M W R Reed. **Role of genetic polymorphisms in tumour angiogenesis.** Br J Cancer. 4; 87(10): 1057–1065 (2002). doi: 10.1038/sj.bjc.6600625	Natalia Prevarskaya, Roman Skryma,Yaroslav Shuba. **Ion Channels in Cancer: Are Cancer Hallmarks Oncochannelopathies?** Physiol. Rev. Vol 98, 2, 559-621 (2018) https://doi.org/10.1152/physrev.00044.2016<br><br>Donna Dang, RajiniRao. **Calcium-ATPases: Gene disorders and dysregulation in cancer.** Biochimica et Biophysica Acta (BBA) - Molecular Cell Research, Vol. 1863, 6, Part B, 1344-1350 (2016) doi.org/10.1016/j.bbamcr.2015.11.016<br><br>Litan A and Langhans SA. **Cancer as a channelopathy: ion channels and pumps in tumor development and progression.** Front. Cell. Neurosci. 9:86. (2015). doi: 10.3389/fncel.2015.00086<br><br>Natalia Prevarskaya, Roman Skryma, Yaroslav Shuba. **Ion channels and the hallmarks of cancer.** Trends Mol Med., 16(3):107-21 (2010). doi: 10.1016/j.molmed.2010.01.005. Epub 2010 Feb 16.		Florence Broders-Bondon, Thanh Huong Nguyen Ho-Bouldoires et al. **Mechanotransduction in tumor progression: The dark side of the force.** J Cell Biol. 217(5), 1571–1587 (2018). doi: 10.1083/jcb.201701039<br><br>LiKang Chin, Yuntao Xia, Dennis E Discher, and Paul A Janmey. **Mechanotransduction in cancer.** Curr Opin Chem Eng. 2016 Feb; 11: 77–84 (2016). doi: 10.1016/j.coche.2016.01.011<br><br>Donald E. Ingber. **Mechanical Signaling and the Cellular Response to Extracellular Matrix in Angiogenesis and Cardiovascular Physiology.** Circulation Research, Vol. 91, 10, 877-887 (2002). doi.org/10.1161/01.RES.0000039537.73816.E5
**Prevention of destruction by the immune system**	S.Vinaya Elizabeth, P.Ryan, Graham Pawelec et al. **Immune evasion in cancer: Mechanistic basis and therapeutic strategies.** Seminars in Cancer Biology, Vol. 35, Suppl. Dec., 185-198 (2015). doi.org/10.1016/j.semcancer.2015.03.004<br><br>H. Gonzalez, C. Hagerling and Z. Werb. **Roles of the immune system in cancer: from tumor initiation to metastatic progression.** Genes & Dev. 2018. 32: 1267-1284 (2018). doi: 10.1101/gad.314617.118<br><br>Alka Bhatia & Yashwant Kumar. **Cellular and molecular mechanisms in cancer immune escape: a comprehensive review**. Expert Review of Clinical Immunology, 10:1, 41-62 (2014). DOI: 10.1586/1744666X.2014.865519	Veronica Huber, Chiara Camisaschi, Angela Berzi et al. **Cancer acidity: An ultimate frontier of tumor immune escape and a novel target of immunomodulation.** Seminars in Cancer Biology, Vol. 43, 74-89 (2017) doi.org/10.1016/j.semcancer.2017.03.001.	Laura Stransky, Kristina Cotter and Michael Forgac. **The Function of V-ATPases in Cancer. Pysiol.** Rev. Vol. 96, 3, 1071-1091 (2016). doi.org/10.1152/physrev.00035.2015	
**Differentiation/ Dedifferentiation**	Park, C.K., Horton, N.C. **Structures, functions, and mechanisms of filament forming enzymes: a renaissance of enzyme filamentation.** Biophys Rev 11, 927–994 (2019). https://doi.org/10.1007/s12551-019-00602-6<br>*Thermodynamics, structure and differentiation*<br><br>Guanghong Wei, Wenhui Xi, Ruth Nussinov, and Buyong Ma. **Protein Ensembles: How Does Nature Harness Thermodynamic Fluctuations for Life? The Diverse Functional Roles of Conformational Ensembles in the Cell.** Chem. Rev. 116, 11, 6516–6551 (2016). doi.org/10.1021/acs.chemrev.5b00562<br><br>Kate Luby-Phelps. **The physical chemistry of cytoplasm and its influence on cell function: an update.** Molecular Biology of the Cell, Vol. 24, No. 17 (2017). doi.org/10.1091/mbc.e12-08-0617<br>*Influence of biophysics on cell function.*<br><br>Aiman Alam-Nazki and J. Krishnan. **Spatial Control of Biochemical Modification Cascades and Pathways.** Biophysical Journal, Vol. 108, 12, 2912-2924 (2015). doi.org/10.1016/j.bpj.2015.05.012<br><br>Vladimir N. Uversky. **Intrinsically Disordered Proteins and Their "Mysterious" (Meta)Physics.** Front. Phys., vol. 7, 10 (2019). doi.org/10.3389/fphy.2019.00010<br>*Significance of IDPs for protein function.*<br><br>Vivek Kulkarnia, Prakash Kulkarnia. **Intrinsically disordered proteins and phenotypic switching: Implications in cancer.** Progress in Molecular Biology and Translational Science, Vol. 166, 63-84 (2019) doi.org/10.1016/bs.pmbts.2019.03.013<br><br>Shachaf, C., Kopelman, A., Arvanitis, C. et al. **MYC inactivation uncovers pluripotent differentiation and tumour dormancy in hepatocellular cancer.** Nature 431, 1112–1117 (2004). https://doi.org/10.1038/nature03043			Humphrey, J., Dufresne, E. & Schwartz, M. **Mechanotransduction and extracellular matrix homeostasis.** Nat Rev Mol Cell Biol 15, 802–812 (2014). https://doi.org/10.1038/nrm3896<br><br>Jordi Alcaraz, Ren Xu, Hidetoshi Mori, Celeste M Nelson, et al. **Laminin and biomimetic extracellular elasticity enhance functional differentiation in mammary epithelia.** EMBO J, 27:2829-2838, (2008) doi.org/10.1038/emboj.2008.206<br><br>A. WayneOrr, Brian P.Helmke, Brett R.Blackman, Martin A.Schwartz. **Mechanisms of Mechanotransduction.** Developmental Cell, Vol. 10, 1, 11-20 (2006). doi.org/10.1016/j.devcel.2005.12.006<br><br>V.M. Weaver,* O.W. Petersen, F. Wang et al. **Reversion of the Malignant Phenotype of Human Breast Cells in Three-Dimensional Culture and In Vivo by Integrin Blocking Antibodies.** JCB, Vol. 137, 1, 231–245 (1997). doi.org/10.1083/jcb.137.1.231<br><br>BL Ricca, G Venugopalan, S Furuta et al. **Transient external force induces phenotypic reversion of malignant epithelial structures via nitric oxide signaling.** eLife 2018;7:e26161 doi: 10.7554/eLife.26161<br><br>Tilghman RW, Cowan CR, Mih JD, Koryakina Y, Gioeli D, Slack-Davis JK, et al. **Matrix Rigidity Regulates Cancer Cell Growth and Cellular Phenotype.** PLoS ONE 5(9): e12905. (201). https://doi.org/10.1371/journal.pone.0012905

Legend: Compilation of publications in which it is shown that the individual forms of cellular work are causally or indirectly involved in the development or manifestation of cancer indicators.
