# Supplementary material for: Origin of Cancer: Cell work is the Key to Understanding Cancer Initiation and Progression
Source: Front Cell Dev Biol. 2022 Mar 1;10:787995. doi: 10.3389/fcell.2022.787995 (PMC8921603; doi:10.3389/fcell.2022.787995)
Supplement: Supplementary file 2 [file DataSheet3.pdf]

# *Supplementary Material*

# Supplement 3: Glossary

**Energy** is a fundamental physical quantity that exists in various forms (potential, kinetic, electrical, chemical, and thermal energy). These forms of energy can be converted into each other. As suggested by Hardin et al. (2012), we would like to use a simplified definition for energy: Energy is the ability to perform change (work). This definition is useful because it characterizes the cell with its essential feature of change and clarifies its total dependence on energy.

Although the above (physical) definition also applies to cells, it is too fundamental to be used in the context of biological and medical processes. For this reason, we use the term biological energy (bioenergy) at certain places to describe the flow of energy in biological mechanisms (Hardin et al., 2012). All life needs a permanent influx of energy to maintain a high degree of order. The cell manages to do this by, for example, taking up chemical energy in the form of nutrients (i.e., glucose), converting it into ATP, GTP (bioenergy), and using it. This provides the cell with the energy required to perform cell work.

**Epigenetic inheritance** refers to inherited traits that are transmitted during mitosis.

**Flow equilibrium**: Many reactions occurring in the body are characterized by flow equilibria. These are characterized by a constant supply of high-energy starting materials and release of low-energy end products. The process is based on the attempt to reach an equilibrium of individual partial reactions, which is never achieved, however, because the products of the reaction are withdrawn by other mechanisms. Thus, the corresponding systems are capable of continuous performance if they are constantly supplied with substances and energy. From this, it can be deduced that life exists far from thermodynamic equilibrium.

**Heat** emitted by the cell is necessary for the fundamental laws of thermodynamics to be met during the working process of the cell or in a defined system. In endothermic animals, it is also important that the organism is kept at a constant temperature (e.g., humans at a core body temperature of 37 °C). The heat required for this is generated by metabolism and, if necessary, by the shivering of the muscles (Hardin et al, 2012). As heat plays a subordinate role in our considerations, it is not discussed in detail here.

**The first law of thermodynamics** states that for any physical or chemical (biological) change, the total amount of energy in the universe remains constant, although the form of energy may change. In other words, energy can be transformed from one form to another, but never created or destroyed. Therefore, it must be stored in systems or released into the environment.

**The second law of thermodynamics states** that with every physical or chemical change, the universe strives to increase disorder or randomness (entropy). This enables us to predict the direction in which a reaction will proceed under specific conditions, how much energy will be released during the reaction, and how specific changes in the conditions will affect the energetics of the reaction.
