# Supplementary material for: Origin of Cancer: Cell work is the Key to Understanding Cancer Initiation and Progression
Source: Front Cell Dev Biol. 2022 Mar 1;10:787995. doi: 10.3389/fcell.2022.787995 (PMC8921603; doi:10.3389/fcell.2022.787995)
Supplement: Supplementary file 3 [file DataSheet1.pdf]

Supplement 1: Arguments against different theories of cancer development.

Mutation theory

<u>Pro:</u>

a. Mutations can be identified in many tumour cells.
b. The existence of familial cancers indicates a causative role of gene mutations in the development of cancer.

<u>Contra:</u>

a. Many gene mutations can also be detected in healthy tissue, including in tumour-relevant genes (e. g. driver genes)[1].
b. Depending on the tumour entity, no mutations in tumour-relevant genes are found in 0-26% of tumours[23].
c. The profiles and number of mutated genes in a tumour are often identical to the surrounding dysplastic tissue of the patient[456].
d. When malignant melanoma cell nuclei are transplanted into an egg, they contribute to the development of a healthy mouse during embryogenesis[7]. As early as 1975, malignant teratocarcinoma cells were transferred into a healthy blastocyst. The chimaera generated from this developed normally and did not show a higher cancer rate[8].
e. The evolution of the species is <u>not</u> based on an increasing number of "beneficial" mutations, but rather the mutations represent an adaptation to recurring changes in the environment[9]. If this fact is taken into account for mutations in cancer cells, they could well be the consequence and not the cause of the disease.

Alternative Theories

Warburg Effect

Otto Warburg postulated as early as the first half of the last century that cancer cells arise from a disturbance in energy production from normal cells. He suggested that two major changes occur as a result of the switch from oxidative phosphorylation to glycolysis. Firstly, the yield of ATP per glucose molecule is reduced and secondly, energy production by glycolysis is rather diffuse, whereas that of oxidative phosphorylation is punctuated. This leads to local changes in the ATP

---

[1] High burden and pervasive positive selection of somatic mutations in normal human skin. Science, Vol 348, No. 6237, p. 880-886, 2015.
[2] Cancer Etiology: Variation in cancer risk among tissues is poorly explained by the number of gene mutations. Genes Chromos Cancer. p. 281-293, 2018.
[3] Somatic Mutation Theory - Why it's Wrong for Most Cancers., Cell Physiol Biochem, 38:1663-1680 (2016). DOI: 10.1159/000443106
[4] Aging and the rise of somatic cancer-associated mutations in normal tissue. PLoS Genet 14(1): e1007108, 2018
[5] Somatic mutant clones colonize the human esophagus with age. Science 362, 911–917, 2018
[6] Genetic alterations in esophageal tissues from squamous dysplasia to cancer. Gastroenterology, 153:166-177, 2017
[7] Reprogramming of a melanoma genome by nuclear transplantation. Genes and Development, 18: 1875-1885, 2004
[8] Normal genetically mosaic mice produced from malignant teratocarcinoma cells. PNAS, Vol. 72, No. 9, p. 3585-589,1975
[9] Connecting cancer to its causes requires the incorporation of effects on tissue microenvironments. Cancer Research, 77(22): 6065-6068, 2017

concentration in the cell. Some cell mechanisms are disturbed in such a way that the result is increasing disorganisation in the cell and malignant transformation[10][11]. According to Warburg, tumour cells always engage in increased glycolysis. This "fermentation" of glucose in the presence of physiological oxygen concentrations is called the Warburg effect or "aerobic glycolysis".

Pro:
The theory is supported by the fact that the vast majority, if not all tumour cells have an altered energy metabolism.

Contra:

a. It has not been proven whether the mechanism described by Warburg underlies the development of all tumours[12]. In fact, it is considered in the textbooks to be a result of the hypoxia or oligemia present in the tumour tissue.
b. Why do patients with a congenital disorder of mitochondrial function not have a higher risk of cancer?[13]? The Warburg hypothesis postulates that a disturbed energy balance causes the development of tumours. As a result, patients with mitochondriopathies should have an increased risk of tumours. However, this is not the case.
c. Doesn't aerobic glycolysis represent a general physiological reaction of metabolism in proliferating cells rather than a tumour-specific mechanism? References to this differentiated view of the Warburg effect are provided by the publication of Vander Heiden[14].
d. Not all tumour cells show a disorder of mitochondria or oxidative phosphorylation. [15]

Disturbed microenvironment

Another theory on cancer initiation and tumour progression is the development of tumours on the basis of a disturbed cell milieu or environment[16][17]. The assumption here is that through the change in the cell environment, for example through inflammation, hypoxia, oligemia, toxins, etc., the cells increasingly change in terms of adaptation, so that ultimately a tumour cell develops.

Pro:

a. Many nonhereditary tumours develop on the basis of chronic inflammation caused, for example, by hypoxia, oligemia, intoxication, radiation or mechanical stimuli[18][19]. These changes are always associated with a change of the milieu. Thus, the cell milieu alone could be responsible for the development of cancer.

---

[10] https://www.mediatheque.lindau-nobel.org/videos/31518/experiments-on-the-chemistry-of-photosynthesis-german-presentation-1954
[11] On the origin of cancer cells. Science, Vol 123, 3191, 1956
[12] The biology of cancer. Second edition, Garland Science, New York and London, 2014
[13] Mitochondrial disease heterogeneity: a prognostic challenge. Acta Myol. 33, 86–93. 2014
[14] Understanding the Warburg Effect: The Metabolic Requirements of Cell Proliferation. Science Vol. 324, p. 1029 – 1033, 2009
[15] Mitochondria and cancer. Nat. Rev. cancer 2012
[16] A microenvironmental model of carcinogenesis. Nature Vol 8, 56-61, 2008
[17] Mina Bissell (LBNL, UC Berkeley): Half the secret of the cell is outside of the cell. https://youtu.be/hBBOMTIXlL0
[18] The biology of cancer. Second edition, Garland Science, New York and London, 2014
[19] Boveri: Concerning the origin of malignant tumours. Translated by Henry Harris, CSHL Press 2008

b. The milieu or external influences can massively influence the phenotype of tumour cells [20] and - strikingly - revert it. The study by Ricca et al. showed that breast cancer cells growing as spheroids changed their growth behaviour to a tubular phenotype *(acinar-like structures*), due to pressure impulses and also displayed reduced cell proliferation[21].
c. Cancer cells in solid tumours are characterised by very high heterogeneity. If the milieu is now significantly involved in the initiation of tumour cells, the theory could explain the massive heterogeneity in the tumour.
d. By cultivating mamma carcinoma cells in an adapted extracellular matrix, they adopt a "normal" growth behaviour[22]

<u>Contra</u>:

a. The theory cannot explain how hereditary cancer syndromes, such as breast, ovarian, hereditary colorectal carcinoma without polyposis (HNPCC) or retinoblastoma develop[23][24].
b. Kaipparettu et al. were able to show that the mere transfer of normal mitochondria into a highly malignant breast cancer cell led to a loss of tumour malignancy and that tumour-specific pathomechanisms were also attenuated[25][26].
c. Is the mutation a consequence of the "unfavourable" environment or is the altered milieu alone the cause of tumour initiation? This question has to date remained unanswered.

TOFT theory

The *Tissue Organization Field Theory* (TOFT-theory) [27][28]assumes that cancer cells arise from a loss of interaction of the cells with their surrounding tissue. This interaction takes place through intercellular "chemical exchange", mechanical forces and bioelectrical interactions. Since these processes play an important role in embryogenesis, the TOFT theory is also called *development gone awry*. The theory assumes that carcinogenesis is characterised by disturbed tissue organisation and high cell proliferation. Three conclusions arise from the TOFT theory[29]:

1. Mutations are not necessary for the development of cancer.
2. Tumours also develop without tissue/cells having previously come into contact with carcinogens.
3. Genetic instability is a byproduct of carcinogenesis.

<u>Pro</u>:

In several experiments it could be shown that after the implantation of tumour cells into a normal healthy stroma tissue, these functioned like normal cells. [30][31]. Conversely, normal cells transplanted into a tumour stroma tissue show the behaviour of cancer cells[32][33].

---

[20] Why don´t we get more cancer? A proposed role of the microenvironment in restraining cancer progression. Nat. Med. 17. 3585-3589. 2011
[21] Transient external force induces phenotypic reversion of malignant epithelial structures via nitric oxide signaling. 7: e26161, eLife. 2018
[22] Laminin signals initiate the reciprocal loop that informs breast specific gene expression and homeostatis by activating NO, p53 and mircoRNAs. eLife 2018
[23] Hallmarks of cancer: the next generation. Cell 144, 646–674. 2011
[24] Cancer etiology. Variations in cancer risk among tissue can be explained by number of stem cell divisions. Science 347, 78–81.
[25] Cancer as a mitochondrial metabolic disease. Front. Cell Dev. Biol. 3, 1–12. 2015
[26] Crosstalk from non-cancerous mitochondria can inhibit tumour properties of metastatic cells by suppressing oncogenic pathways. PLoS ONE 8:e61747.
[27] The somatic mutation theory of cancer: growing problems with the paradigm? Bioassays, 26(10), 1097-1107, 2004
[28] The society of Cells: Cancer and control of cell proliferation. New York Springer Verlag, 1999
[29] A Cancer theory kerfuffle can lead to new Lines of Research. J Natl. Cancer Inst. 107(2), dju 405, 2015
[30] Reprogramming human cancer cells in the mouse mammary gland. Cancer Research, 70 (15), 6336-6343, 2010
[31] Totipotency and normal differentiation of single teratocarcinoma cells cloned by injection into blastocytes. PNAS 73(2), 549-553, 1976
[32] Development of tumours in the rat ovary after transplantation into the spleen. Proc. Soc. Exp. Biol. Med. 55(3), 176-179, 1944
[33] The stroma as a crucial target in rat mammary gland carcinogenesis. J Cell Sci, 117, 1495-1502, 2004

Contra:
If the transfer of functionally normal mitochondria leads to the loss of malignancy of a tumour cell,[34] the TOFT theory cannot explain this phenomenon, as it considers the loss of cell/cell and cell/extracellular matrix contacts as the cause.

Speciation theory
The theory is that the first step in the development of cancer is characterised by numerical and/or structural changes in the chromosomes. Depending on the type of change, they show a massive loss or gain of DNA/genes, which leads to an imbalance. Eventually, a cancer cell can develop from this.

Pro: Many solid tumours are aneuploid. In addition, precancerous lesions (dysplasias) or inflammatory tissue are characterised by chromosomal changes and instability[35][36][37][38].

Contra:

a. Aneuploidy and chromosomal aberrations are irreversible changes which, according to this theory, are responsible for the initiation and progression of tumours. But how does the theory then, explain the loss of malignancy in tumour cells when healthy mitochondria have been injected into them?[37]
b. Not every tumour shows chromosomal changes[39][40].
c. Constitutional aneuploidies can be identified in the brain[41]. Based on the aneuploidy hypothesis, it could be concluded that these patients would have an increased risk of developing a brain tumour. However, this is not the case.
d. Patients with constitutional trisomy 21 show an increased incidence of leukaemia, but they have a 50% lower risk of developing solid tumours[42][43].

[34] Crosstalk from non-cancerous mitochondria can inhibit tumour properties of metastatic cells by suppressing oncogenic pathways. PLoS ONE 8:e61747.
[35] Origin of Cancer: An Information, Energy, and Matter Disease. Front. Cell Dev. Biol., November 2016
[36] Hallmarks of cancer: the next generation. Cell 144, 646–674, 2011
[37] Does aneuploidy destabilize karyotypes automatically? Proc. Natl. Acad. Sci. U.S.A. 111:E974
[38] Correlation between dysplasia and ploidy status in oral leukoplakia. Head Neck Pathol. 6, 322–327, 2012
[39] Genetic instability in human cancer. Nature 396, 643–649. 1998
[40] The biology of cancer. Second edition, Garland Science, New York and London, 2014
[41] Constitutional aneuploidy in the normal human brain. J. Neurosci. 25, 2176–2180. 2005
[42] Risks of leukaemia and solid tumours in individuals with Down's syndrome. Lancet 355, 165–169, 2000
[43] Down's syndrome suppression of tumour growth and the role of the calcineurin inhibitor DSCR1. Nature 20 May 2009
